# Foliar and Seed Application of Amino Acids Affects the Antioxidant Metabolism of the Soybean Crop

**DOI:** 10.3389/fpls.2017.00327

**Published:** 2017-03-21

**Authors:** Walquíria F. Teixeira, Evandro B. Fagan, Luís H. Soares, Renan C. Umburanas, Klaus Reichardt, Durval D. Neto

**Affiliations:** ^1^Department of Crop Science, “Luiz de Queiroz” College of Agriculture, University of São PauloPiracicaba, Brazil; ^2^Department of Agronomy, University Center of Patos de MinasPatos de Minas, Brazil; ^3^Center for Nuclear Energy in Agriculture, University of São PauloPiracicaba, Brazil

**Keywords:** glutamate, cysteine, phenylalanine, glycine, *Glycine max*

## Abstract

In recent years, the application of natural substances on crops has been intensified in order to increase the resistance and yield of the soybean crop. Among these products are included plant biostimulants that may contain algae extracts, amino acids, and plant regulators in their composition. However, there is little information on the isolated effect of each of these constituents. The objective of this research was to evaluate the effect of the application of isolated amino acids on the antioxidant metabolism of the soybean crop. Experiments were carried out in a greenhouse and in the field with the application of the amino acids glutamate, phenylalanine, cysteine, glycine in seed treatment, and foliar application at V_4_ growth stage. Antioxidant metabolism constituents evaluated were superoxide dismutase, catalase, peroxidase, hydrogen peroxide content, proline, and lipid peroxidation. In addition, resistance enzymes as polyphenol oxidase and phenylalanine ammonia-lyase (PAL) were evaluated. In both experiments, the use of cysteine, only in seed treatment and in both seed treatment and foliar application increased the activity of the enzyme PAL and catalase. Also in both experiments, the use of phenylalanine increased the activity of the enzyme PAL when the application was carried out as foliar application or both in seed treatment and foliar application. In the field experiment, the application of glutamate led to an increase in the activity of the catalase and PAL enzymes for seed treatment and foliar application. The use of the set of amino acids was only efficient in foliar application, which led to a greater activity of the enzymes peroxidase, PAL, and polyphenol oxidase. The other enzymes as well as lipid peroxidation and hydrogen peroxide presented different results according to the experiment. Therefore, glutamate, cysteine, phenylalanine, and glycine can act as signaling amino acids in soybean plants, since small doses are enough to increase the activity of the antioxidant enzymes.

## Introduction

Soybean [*Glycine max* (L.) Merr.] grains are consumed at large scale with different purposes. One of them is the production of soybean oil for human consumption. It is also used as feed, flour, soap, cosmetics, resins, paints, solvents, and biodiesel, mainly (Kim et al., 2016). In order to increase crop yield, the use of biostimulants, both as seed treatment (ST) and foliar application (FA), is promising (Khan et al., 2009; Craingie, 2011; Soares et al., 2016). These products can increase the initial vigor of plants, and can thus increase resistance against diseases or other types of stress, which can increase yield (Lucini et al., 2015). Plant biostimulants are derived especially from algae extracts, protein hydrolysates, and humic substances (Battacharyya et al., 2015; Canellas et al., 2015; Colla et al., 2015).

Amino acids are organic molecules that contain nitrogen, carbon, hydrogen, and oxygen, and have an organic side-chain in their structure, a characteristic that distinguishes the different amino acids (Buchanan et al., 2000). The main amino acids synthesized by plants are the glutamate, glutamine, and aspartate, and from these other amino acids may be formed. Glutamate stands out for being the first amino acid in which the nitrogen absorbed by the plants is incorporated and from it, a range of amino acids can be obtained through the activity of aminotransferases (Buchanan et al., 2000; Taiz and Zeiger, 2013).

Amino acids can play different roles in plants; they can act as stress-reducing agents, source of nitrogen and hormone precursors (Zhao, 2010; DeLille et al., 2011; Maeda and Dudareva, 2012). In the soil, they can be found in different forms, however, their half-life is short and their absorption by plants is only possible due to the presence of transporters in the roots (Jamtgard et al., 2010).

Several studies have shown the efficiency of amino acids uptake by plants (Persson et al., 2006; Gioseffi et al., 2012). In this context, the application of amino acids via seed can result in a better plant development, since these molecules can act as signals of several beneficial physiological processes of plants. Studies show that FA of amino acids in plants is promising (Abdel-Mawgoud et al., 2011; Koukounaras et al., 2013; Sadak et al., 2014), but the effect of the application of amino acids on the oxidative metabolism of soybean has received little attention.

Despite the knowledge about the positive effect of amino acid application on plants, most of the studies were carried out with products composed of a set of amino acids, and there is little information regarding the isolated effect of these amino acids on plants (Khan et al., 2009; Colla et al., 2014). In addition, amino acids such as glutamate, cysteine, phenylalanine, and glycine may act directly or indirectly in the attenuation of plant oxidative stresses (Denisov and Afanas’Ev, 2005; Ashraf and Foolad, 2007; Gill and Tuteja, 2010). Thus, their application on seeds or leaves may be an alternative to attenuate the effects caused by the oxidative stress that plants may suffer.

The present work aimed to evaluate the effect of glutamate, cysteine, phenylalanine, and glycine application (via seeds, via leaves or both cases) on the oxidative metabolism of the soybean crop.

## Materials and Methods

In greenhouse and field experiments, the amino acid treatments (glutamate, phenylalanine, cysteine, glycine and all these amino acids in association) were applied on the seeds (ST), or on the leaves at the V_4_ growth stage (FA) or both – (ST + FA). An control treatment where only water was applied on seeds and leaves was also included in the experiments.

### Greenhouse Experiment

The soybean (*Glycine max* [L.] Merrill – cultivar NS 7901 RR) greenhouse experiment was carried out (sowing date: September 10, 2015) at ‘Piracicaba,’ São Paulo State, Brazil (22°42′ South, 47°38′ West, altitude of 546 m) using 11 dm^3^ pots, containing washed sand as substrate. Ten seeds were sown per pot and after emergence thinned to three plants, following a randomized block design with eight replications.

Amino acid applications were performed as ST, FA at V_4_ growth stage (Fehr and Caviness, 1977), or ST and FA.

In the ST, the following amino acids were applied: (i) glutamate (Glu): 10.5 mM, (ii) cysteine (Cys): 25.524 mM, (iii) phenylalanine (Phe): 4.676 mM, (iv) glycine (Gly) 30.904 mM, and (v) Glu + Cys + Phe + Gly: 10.5 mM + 25.524 mM + 4.676 mM + 30.904 mM, respectively.

In the FA, the following amino acids were applied: (i) Glu: 4.2 μM, (ii) Cys: 5.15 μM, (iii) Phe: 0.94 μM, (iv) Gly: 6.18 μM, and (v) Glu + Cys + Phe + Gly: 4.20 μM + 5.15 μM + 0.94 μM + 6.18 μM, respectively.

The control treatment received only with water, both in ST and FA. The concentrations of amino acids were determined based on tests carried out previously (**Table 1**).

**Table 1:** Concentrations of different amino acids applied on seeds [seed treatment (ST)], leaves at V_4_ crop growth stage [foliar application (FA)], or on both ST and FA.

Amino acid treatments	ST (mg kg^-1^ [seeds])	FA (mg ha^-1^)	ST (mg kg^-1^ [seeds]) + FA (mg ha^-1^)	
Control	0	0	0	0
Glutamate (Glu)	12	123	12	123
Cysteine (Cys)	12	123	12	123
Phenylalanine (Phe)	3	30	3	30
Glycine (Gly)	9	92	9	92
Glu + Cys + Phe + Gly	12 + 12 + 3 + 9	123 + 123 + 30 + 92	12 + 12 + 3 + 9	123 + 123 + 30 + 92

For the ST, the amino acids were diluted in water and applied in the rate of 1 mL kg^-1^ of seeds with the respective concentrations shown in **Table 1**. The amino acid solution was applied to the seeds, which were placed in plastic bags, and homogenized by friction. On the same day, the sowing was carried out. The sources used correspond to the pure amino acids, with levogyrous (L-amino acid) optical isomerism.

Foliar application of amino acid treatments were applied at the V_4_ growth stage with a CO_2_ pressurized backpack sprayer, which delivered 200 L ha^-1^ at 200 kPa. Before ST application, all seeds were treated with fungicide and insecticide (Fipronil [250 g L^-1^], Methyl Thiophanate [225 g L^-1^], and Pyraclostrobin [25 g L^-1^]) at a rate of 1 mL kg^-1^ of seeds. The pots were irrigated daily according to water requirement (400 mL per pot), and weekly a nutrient solution was applied as proposed by Johnson et al. (1957).

### Field Experiment

Soybean seeds [*Glycine max* (L.) Merrill; cv NS 7901 RR] were sown on January 26, 2014 and conducted on an experimental area in Patos de Minas, Minas Gerais State, Brazil (18°34′ S, 46°31′ W, alt. 815 m).

The soil of the site is classified as an Oxisol (Soil Survey Staff, 2014) and the area presents a tropical climate of altitude (Cwa) according to Köppen, with annual average precipitation around 1,400 mm (Souza et al., 2005).

Based on soil analysis, 36 kg ha^-1^ of N, 54.6 kg ha^-1^ of P, 37.5 kg ha^-1^ of K, 10.5 kg ha^-1^ of Ca, 19.1 kg ha^-1^ of S, 0.9 kg ha^-1^ of B and 0.9 kg ha^-1^ of Zn were applied before sowing.

For weed control, the herbicide Glyphosate was applied at 17 and 32 days after sowing (DAS) (650 g [a.i.] U^-1^ at a rate of 2.2 kg [b.w.] ha^-1^). For insect control, it was used Methomyl (215 g [a.i.] ha^-1^ at a dose of 1.5 U [b.w.] ha^-1^) at 80 and 106 DAS, and Methamidophos (600 g [a.i.] L^-1^ at a rate of 1.0 L [p.c.] ha^-1^) at 90 DAS. For disease control, Pyraclostrobin and Epoxiconazole were used (133 g [a.i.] ha^-1^, 50 g [a.i.] ha^-1^, respectively, at a dose of 0.6 L [p.c.] ha^-1^), and Carbendazim (663 g [a.i.] U^-1^ at a dose of 0.6 U [b.w.] ha^-1^) at 90 and 106 DAS.

The statistical design comprised randomized blocks with eight replications, using the same treatments as those of the greenhouse experiment.

Each plot was composed of four lines 7 meters long, 0.45 m between rows, totalizing a useful area of 4.5 m^2^. Soybean plant density was 250,000 plants ha^-1^.

Seed treatment and foliar application with amino acids were performed in the same way as in the greenhouse experiment.

### Biochemical Determinations

In experiment I, plant samples were hand-harvested for biochemical determinations from ST at stages V_3_ and V_6_, and from FA only at stage V_6._ In experiment II, plant samples were hand-harvested from ST and FA only at stage V_6_. In each evaluation, were performed eight replicates each consisting of four pooled plants.

Samples of the fresh leaf biomass were ground, and 200 mg were macerated with 4 mL of 0.1 mol L^-1^ potassium phosphate buffer pH 6.8. Samples were transferred to Eppendorf flasks and centrifuged at 10,000 rpm for 30 min at 4°C (Kar and Mishra, 1976). At the end, they were stored at -20°C until determinations of leaf protein content (Bradford, 1976), superoxide dismutase (SOD) activity (Beauchamp and Fridovich, 1971), catalase (CAT) activity (Peixoto et al., 1999), peroxidase (POD) activity (Teisseire and Guy, 2000), phenylalanine ammonia-lyase (PAL) activity (Umesha, 2006), and polyphenol oxidase (PPO) activity (Duangmal and Apenten, 1999). From the fresh leaf biomass were also analyzed hydrogen peroxide content, (H_2_O_2_) (Alexieva et al., 2001), lipid peroxidation (LP) content (Heath and Packer, 1968), and proline content (Bates et al., 1973).

### Statistical Analysis

Data of the two experiments were evaluated for normality and homogeneity using the Shapiro–Wilk and Levene tests, respectively, both at the 5% significance level. A variance analysis was performed and, when significant, the Duncan test was applied at the 5% level of significance.

For the field experiment, a multivariate analysis was performed through Principal Component Analysis. All analyses were performed using the statistical software SAS 9.3 (Sas Institute, 2011).

## Results

### Greenhouse Experiment

#### Crop Response at the V_3_ Growth Stage

**FIGURE 1:**
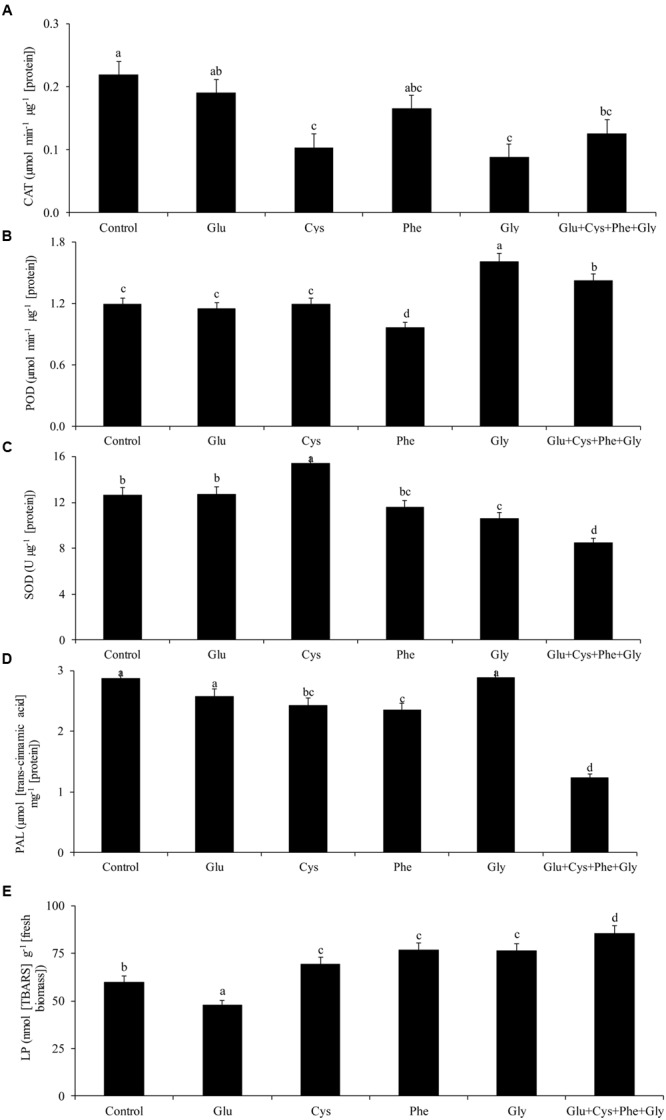
**Activity of the enzymes catalase (CAT, A**), peroxidase (POD, **B**), superoxide dismutase (SOD, **C**), phenylalanine ammonia-lyase (PAL, **D**), and lipid peroxidation (LP, **E**), in soybean plants at V_3_ growth stage subjected to treatments with amino acids as seed treatment (ST) in the greenhouse experiment. Means followed by the same lowercase letters do not differ significantly from each other, using the Duncan test at 5% significance.

The application of amino acids in ST reduced the activity of PAL, except in the case of glycine and glutamate treatments (**Figure 1D**).

The activity of the enzyme PAL was not affected by the application of amino acids (**Figure 1D**). However, glycine application increased POD activity by 35%, in relation to the control (**Figure 1C**).

Glycine application also increased POD activity by 35%, in relation to the control (**Figure 1C**).

The lowest LP occurred with glutamate application (**Figure 1E**). This treatment did not increase the activity of CAT, POD, and SOD (**Figures 1A,B,C**, respectively), which shows that the plant used other mechanisms to reduce free radicals and, consequently, to reduce LP.

The application of cysteine increased the SOD activity by 21% in relation to the control, while the joint application of the glutamate, cysteine, phenylalanine, and glycine (Glu + Cys + Phe + Gly) reduced the SOD activity (**Figure 1C**).

Glu + Cys + Phe + Gly increased by 43% the LP in relation to the control (**Figure 1E**).

Glycine applied only in FA increased SOD activity in relation to the control (**Figure 1C**), as well as glutamate and cysteine when applied in ST and FA.

#### Crop Response at V_6_ Growth Stage

**FIGURE 2:**
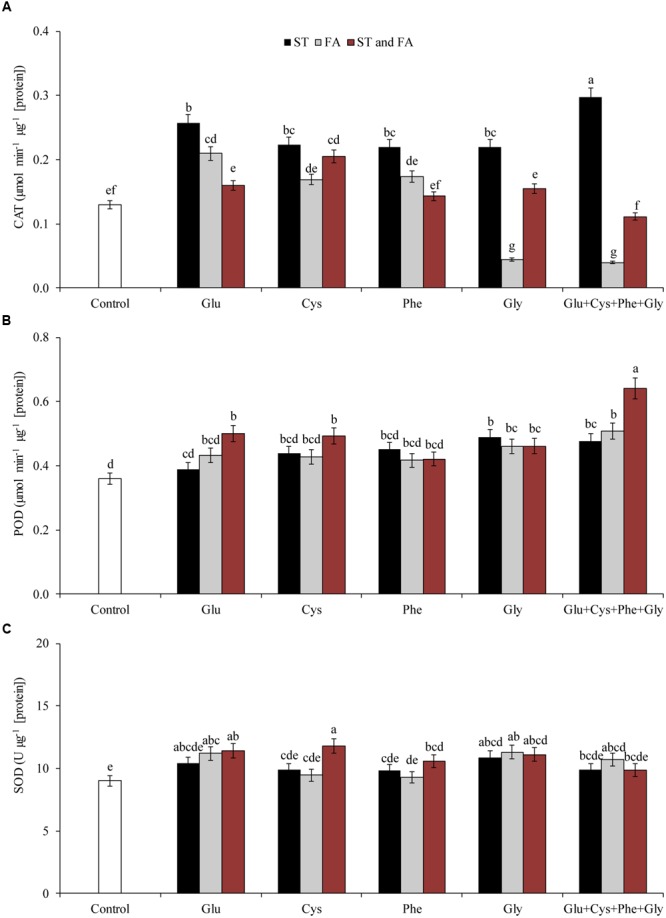
**Activity of the enzymes CAT (A)**, POD **(B)**, and SOD **(C)**, in soybean plants at V_6_ growth stage submitted to amino acid application as ST and as foliar application (FA) at V_4_ or both in the greenhouse experiment. Means followed by the same lowercase letters do not differ significantly from each other, using the Duncan test at 5% significance.

Glu + Cys + Phe + Gly applied in ST was the treatment that increased most the activity of the enzyme CAT, by 128% in relation to the control (**Figure 2A**). The other treatments also increased the activity of this enzyme, except glycine applied in FA and Glu + Cys + Phe + Gly applied in both ST and FA. However, Glu + Cys + Phe + Gly applied in both ST and FA increased the POD activity (**Figure 2B**).

**FIGURE 3:**
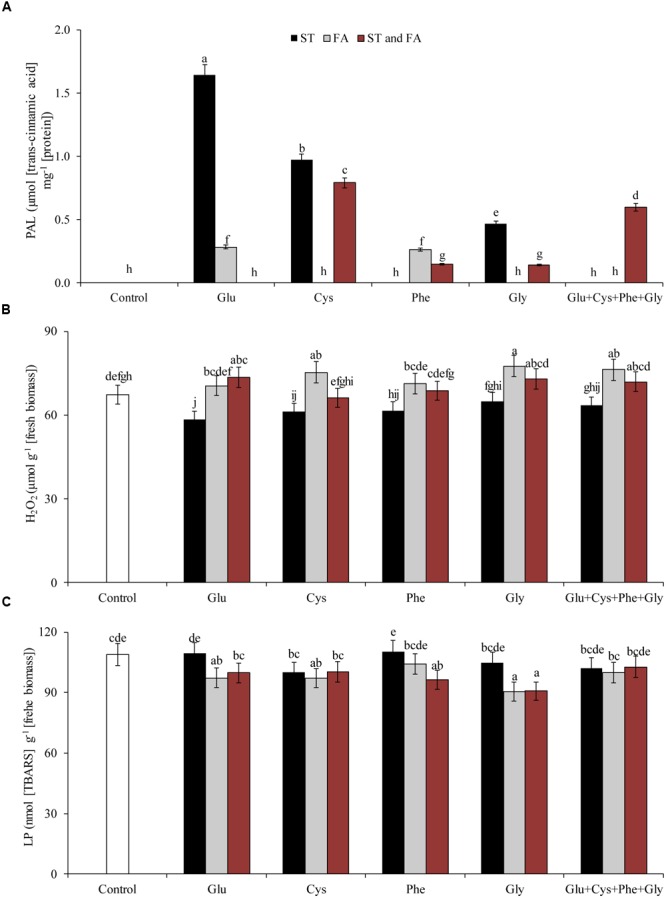
**Activity of the enzyme PAL (A)**, hydrogen peroxide content (H_2_O_2_, **B**), and LP **(C)**, in soybean plants at V_6_ growth stage submitted to amino acid application as ST and as FA at V_4_ or both in the greenhouse experiment. Means followed by the same lowercase letters do not differ significantly from each other, using the Duncan test at 5% significance.

Glutamate applied in ST reduced the H_2_O_2_ content by 13% in relation to the control, however, glycine in FA increased H_2_O_2_ content by 15% in relation to the control (**Figure 3B**).

Glutamate applied in ST was the treatment that most increased PAL activity (**Figure 3A**). Cysteine and glycine applied in ST, glutamate and phenylalanine applied in FA, and Glu + Cys + Phe + Gly applied in both ST and FA, increased the PAL activity by at least 47% (**Figure 3A**).

Amino acid applied in ST did not increase LP, but glycine applied in FA and in both ST and FA, reduced LP by 18% in relation to the control (**Figure 3C**).

The only amino acid that had consistent effects at both stages was glycine, which increased the activity of POD and PAL. However, it was not possible to observe a response pattern for other treatments in both evaluation times (V_3_ and V_6_).

### Field Experiment

#### Crop Response at V_6_ Growth Stage

**FIGURE 4:**
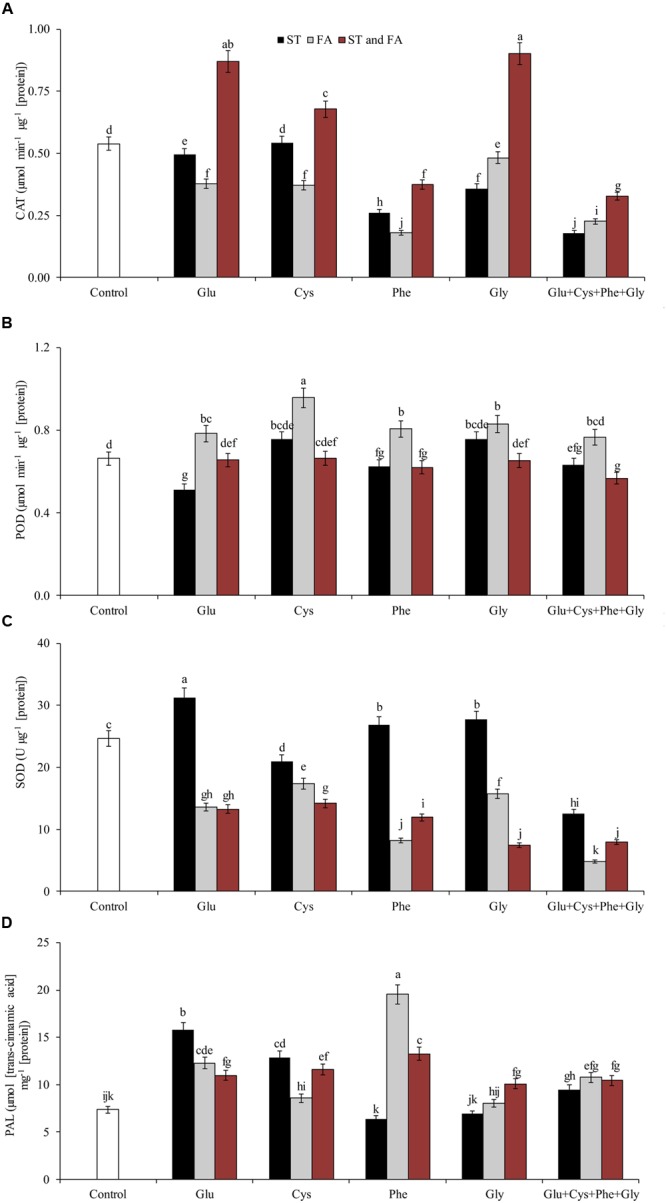
**Activity of the enzymes CAT (A)**, POD **(B)**, SOD **(C)**, and PAL **(D)** in soybean plants at V_6_ growth stage submitted to amino acid application as ST and as FA at V_4_ or both in the field experiment. Means followed by the same lowercase letters do not differ significantly from each other, using the Duncan test at 5% significance.

Amino acid application altered enzyme activity in soybean plants. Glycine when applied both in ST and FA increased CAT activity by 67% in relation to the control (**Figure 4A**). Glutamate and cysteine in ST and FA increased CAT activity by 62 and 26%, respectively. Cysteine in FA increased POD activity by 45% in relation to the control (**Figure 4B**).

Glutamate applied in ST increased SOD activity by 27% in relation to control (**Figure 4C**). In general, the application of amino acids in ST was more effective increasing SOD activity.

**FIGURE 5:**
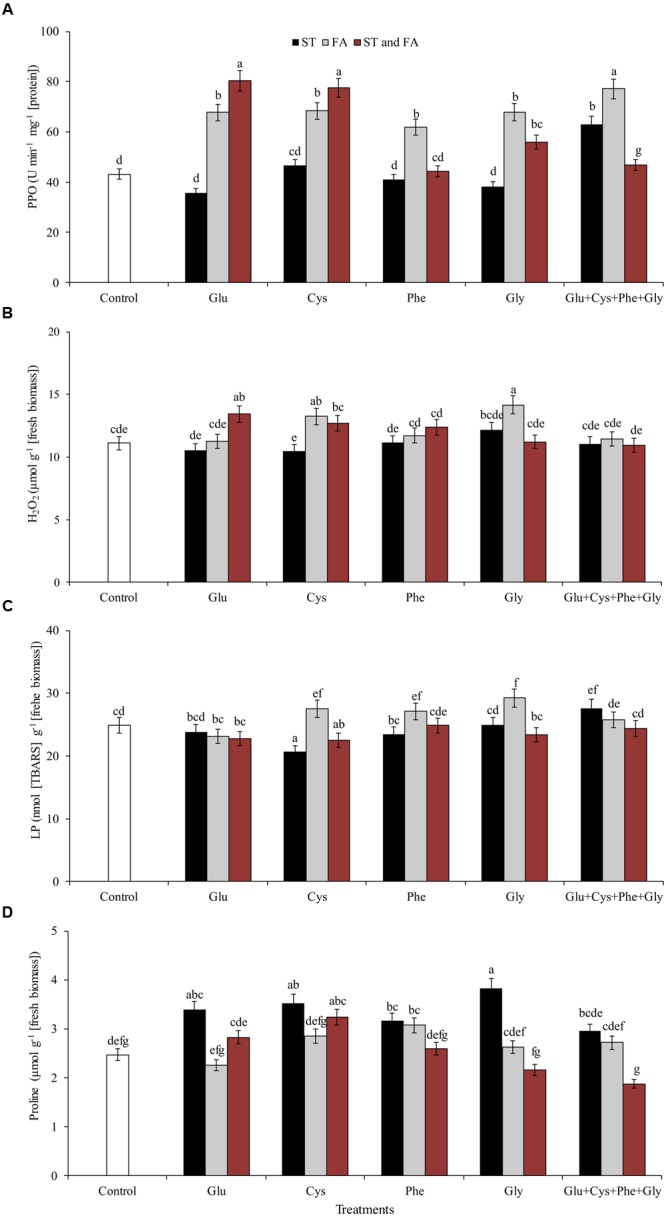
**Activity of the enzymes polyphenol oxidase (PPO, A)**, hydrogen peroxide content (H_2_O_2_, B), LP **(C)**, and proline content **(D)**, in soybean plants at V_6_ growth stage submitted to amino acid application as ST and as FA at V_4_ or both in the field experiment. Means followed by the same lowercase letters do not differ significantly from each other, using the Duncan test at 5% significance.

Amino acid application also affected the activity of PAL (**Figure 4D**) and PPO (**Figure 5A**) enzymes. Phenylalanine (applied in FA) and glutamate (applied in ST) increased the activity of PAL enzyme by 165 and 114%, respectively, in relation to the control. The other treatments, except phenylalanine and glycine applied in ST and cysteine and glycine in FA, increased the PAL activity by at least 46% in relation to the control.

Glu + Cys + Phe + Gly in FA, glutamate and cysteine in ST and FA were the treatments that most increased the PPO enzyme activity, by 78, 85, and 79%, respectively, in relation to the control. The application of glutamate, phenylalanine, and glycine in FA also increased PPO activity, but in lower intensity (**Figure 5A**).

Glycine applied as ST increased the proline content by 55% in relation to the control (**Figure 5D**). Glycine in FA increased the H_2_O_2_ content by 9% in relation to the control (**Figure 5B**). This same amino acid in ST or FA also increased SOD activity (**Figure 4C**), which may have resulted in the increase of H_2_O_2_ contents, as this enzyme dissociates O_2_^-^ into H_2_O_2_ and H_2_O. Due to the increase in free radicals (H_2_O_2_), there was also an increase in LP (**Figure 5C**). Cysteine applied in ST reduced LP by 21% in relation to the control. On the other hand, the use of glycine in FA increased the LP by 17% in relation to the control (**Figure 5C**).

**FIGURE 6:**
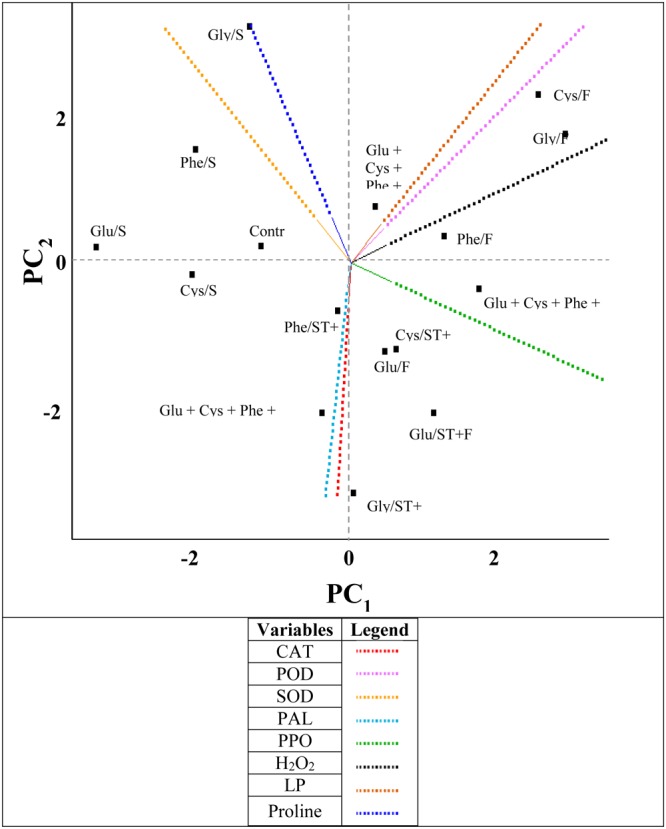
**Byplot obtained from the principal components analysis (PC) of the results of the variables (CAT, catalase; POD, peroxidase; SOD, superoxide dismutase; PAL, phenylalanine ammonium-lyase; PPO, polyphenol oxidase; H_2_O_2_, hydrogen peroxide; LP, lipid peroxidation and the proline were evaluated in the soybean crop submitted to the application of amino acids as ST (Glu/ST, Glutamate; Cys/ST, Cysteine; Phe/ST, Phenylalanine; Gly/ST, Glycine; Glu + Cys + Phe + Gly/ST); as FA: (Glu/FA, Glutamate; Cys/FA, Cysteine; Phe/FA, Phenylalanine; Gly/FA, Glycine; Glu + Cys + Phe + Gly/FA) and applied as ST and as FA (Glu/ST + FA, Glutamate; Cys/ST + FA, Cysteine; Phe/ST + FA, Phenylalanine; Gly/ST + FA, Glycine; Glu + Cys + Phe + Gly/ST + FA)**.

When the amino acids were applied as ST, in general the proline content and SOD enzyme activity increased and LP content was reduced (**Figure 6**). On the other hand, the evaluated amino acids in FA increased the H_2_O_2_ and LP contents.

The amino acids applied as ST and as FA increased the activity of the enzymes PAL, CAT, and PPO (**Figure 6**). A positive correlation between the CAT and PAL enzymes could be observed, and between the LP and the H_2_O_2_ content. In addition, there was a negative correlation between CAT and PPO.

The variables that presented more percentage of variance were PPO and proline, which represented the principal components PC_1_ and PC_2_, respectively (**Table 2**).

**Table 2:** Eigen values, percentage of variance, cumulative variance, eigen vectors for different principal components.

Principal component (PC)	Eigen values	Percentage of variance	Cumulative variance	Eigen vectors		
				Variable	PC_1_	PC_2_
1	2.62	0.53	0.53	CAT	–0.03	–0.42
2	1.69	0.21	0.74	POD	0.41	0.34
3	1.42	0.13	0.87	SOD	–0.41	0.44^1^
4	1.08	0.06	0.93	PAL	–0.03	–0.26
5	0.53	0.03	0.96	PPO	0.50^1^	–0.20
6	0.36	0.02	0.98	H_2_O_2_	0.44^1^	0.18
7	0.16	0.01	0.99	PL	0.37	0.40
8	0.14	0.01	1.00	Proline	–0.23	0.46^1^

*^1^Values with higher weights within PC.*

**FIGURE 7:**
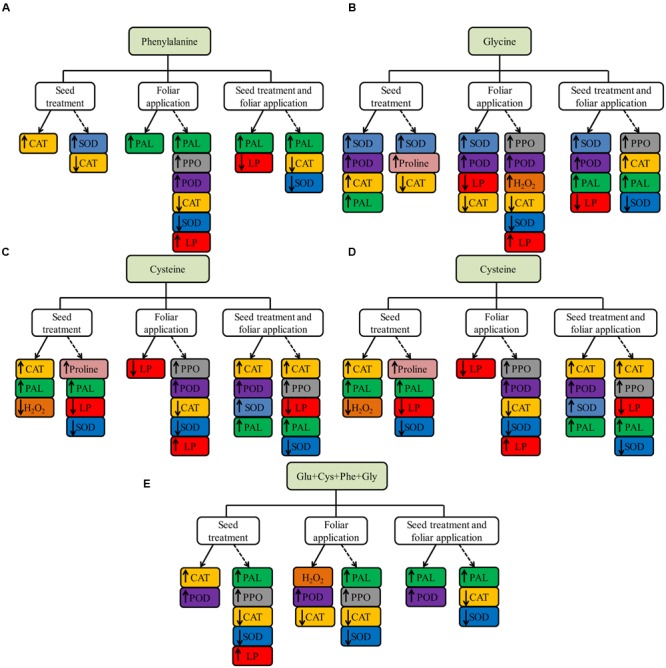
**Summary of the effect of phenylalanine (Phe, A)**, glycine (Gly, **B**), cysteine (Cys, **C**), glutamate (Glu, **D**) and set of amino acids (Glu + Cys + Phe + Gly, **E**), applied in the ST, foliar application and both, in soybean crop in greenhouse experiment (continuous line) and field experiment (dotted line), evaluations carried out in V_6_ stage.

The results of amino acid effects on enzyme activities and proline, both of greenhouse and field experiments, are summarized in **Figure 7**.

## Discussion

The plant can use the amino acids in different signaling pathways according to their stage of development. This may be the reason why amino acid responses, with the exception of glycine, were not consistent at both evaluation times (V_3_ and V_6_) in greenhouse experiment.

Amino acids are important components of antioxidant systems in plants. The action of these molecules involves the reduction of free radicals and osmoprotection (Ashraf and Foolad, 2007; Gill and Tuteja, 2010; Rennenberg and Herschbach, 2014). In addition, amino acids play a key role in signaling stress response and secondary metabolism in plants (Hildebrandt et al., 2015). Glycine acts on the stress response because it forms glycine betaine, a compatible solute that acts as an osmoprotector in plants, especially when submitted to salt stress conditions (Demiral and Turkan, 2006).

From the production of glycine betaine, several signaling processes start in plants, such as increased activity of antioxidant enzymes and the consequent reduction of LP (Hu et al., 2012). These events were observed in this study in the greenhouse experiment, where glycine applied in ST increased SOD activity (**Figure 2C**). The use of glycine in FA increased PPO and POD activities in both experiments. The application of glycine can directly increase or indirectly signaling the production of glycine betaine, which acts on the signaling mechanism of the antioxidant and defense system, increasing the activity of the enzymes.

On the other hand, glycine is also involved in the route of glyoxylate production, a compound that can reduce H_2_O_2_ content in plants, leading to the reduction of LP (Alhasawi et al., 2015). All these events occurred in the greenhouse experiment with FA of glycine (**Figures 3A,B**). In addition, glyoxylate can produce NADPH and ATP, energy molecules used in various metabolic processes (Alhasawi et al., 2015).

Analyzing the spectrum of antioxidant action, cysteine is considered a key amino acid. It can act in the antioxidant metabolism directly through the production of phytochelatins, which help in the control of metal excess in plants, and it is related in the production of glutathione (GSH), molecules that regulate the production of free radicals (Cobbett, 2000). In addition, some studies have shown that cysteine also acts as a signal to increase the activity of antioxidant enzymes and reduction of LP (Azarakhsh et al., 2015).

Azarakhsh et al. (2015) evaluated the effects of FA (vegetative phase) and via STs of cysteine rates on basil and observed that the concentration of 2.5 mM cysteine (61.8 g ha^-1^) promoted reduction of LP in the two application moments. The use of the same dose as ST also promoted an increase in the activity of CAT and PAL enzymes. However, in our experiment it was possible to obtain similar results using rates lower than those used in Azarakhsh et al. (2015). The use of cysteine at a rate of 123.7 mg ha^-1^, as FA, promoted the increase of CAT activity (**Figure 2A**) and reduction of LP (**Figure 3B**) in the greenhouse experiment. In the field experiment, this result was observed with the use of cysteine in both ST and FA applications. In this way, the application of this amino acid can increase the activity of antioxidants and PAL enzymes. This fact was observed when this amino acid was applied as ST, in both experiments.

In the same way that glycine and cysteine acted on oxidative stress, the use of phenylalanine was also promising. This amino acid is involved in biosynthetic routes of secondary metabolism. Various substances can be synthesized from phenylalanine, such as phenolic compounds, among them flavonoids and lignin. The reaction for the production of these compounds is made from the enzyme PAL, which catalyzes phenylalanine, producing cinnamic acid that leads to the production of flavonoids or lignin (Taiz and Zeiger, 2013).

Lignin provides several benefits for plants such as mechanical strength, mainly in the stem and vascular tissues, which determines a greater plant growth and favors the conduction of water and minerals through the xylem (Taiz and Zeiger, 2013). In this work, the use of phenylalanine applied in the vegetative phase had an increase in the activity of PAL in greenhouse experiment (**Figure 3A**) and field experiment (**Figure 4D**), which indicates, therefore, that the use of this amino acid may have an indirect effect on the improvement of the secondary metabolism of plants.

Application of the Glu + Cys + Phe + Gly applied both in ST and FA provided increment of PAL in the greenhouse (**Figure 3A**) and field (**Figure 4D**) experiments. This evidences that there may have been a cumulative effect of the action of these amino acids, because when applied in an isolated way as ST, they did not affect the defense metabolism.

Some amino acids such as glutamate can act to attenuate oxidative stress indirectly by being the precursor of other amino acids such as arginine and proline, which are related to the reduction of plant stress (Gill and Tuteja, 2010; Rejeb et al., 2014).

In addition, glutamate is involved in the production of GSH, a compound that decreases plant stress by binding to some free radicals, stabilizing its negative effect on plants, and is also used as a substrate for some enzymes responsible for oxidative metabolisms such as glutathione peroxidase and glutathione synthetase (Mittler, 2002; Gill and Tuteja, 2010).

In spite of these beneficial characteristics of glutamate, the reduction of the oxidative stress evaluated by LP was not evidenced, although some important enzymes of the oxidative metabolism were benefited by the application as ST and FA of this amino acid, such as CAT (**Figure 2A**). In addition, the use of this amino acid promoted the increase of H_2_O_2_ content in leaves of the greenhouse experiment.

Another indirect effect of glutamate application may have occurred when it increased the activity of PAL (**Figures 3A**, **4D**), because glutamate is a precursor of arginine, and this amino acid can increase PAL activity (Zheng et al., 2011). This process occurs because arginine is a precursor of polyamines, compounds that can act as defense signal in plants (Kuznetsov et al., 2006). In addition, polyamines may induce increased activity of antioxidant enzymes (Shi et al., 2010; Liu et al., 2015). This explains the fact that the application of glutamate increased the activity of SOD and CAT, due to its indirect effect.

In addition to the direct effects of amino acids on the metabolisms discussed above, were revealed in plants the existence of glutamate receptors (GLR) homologous to those in animals (Price et al., 2012). In *Arabidopsis thaliana*, 20 GLR genes were characterized (Forde and Roberts, 2014).

Studies using Arabidopsis (*Arabidopsis thaliana* (L.) Heynh.) GLR knockout mutants have indicated that GLR receptors are gated by a broad spectrum of amino acids, including L-glutamate, L-serine, L-alanine, methionine, tryptophan, phenylalanine, leucine, asparagine, threonine, cysteine, glycine, tyrosine, and peptides such as GSH (Vincill et al., 2012; Forde and Roberts, 2014).

Based on this information, it is shown that amino acids activate rapid responses when applied exogenously in plants (Price et al., 2012). The application of amino acids can increase the concentration of Ca^2+^ in the cytoplasm and depolarize the membrane (Qi et al., 2006; Stephens et al., 2008). In addition GLRs can mediate physiological effects such as root architecture, plant development, phototropism, gravitropism, plant stress signaling, abscisic stress signaling, carbon metabolism, stomatal movements, photosynthesis, and plant immunity (Weiland et al., 2015).

Foliar application of amino acids Glu, Cys, Phe, and Gly, alone or in combination, increased PPO activity (**Figure 5A**). These amino acids play an important role in signaling in plants, which may increase the activity of antioxidant enzymes and resistance enzymes such as PPO.

A PPO plays an important role in plants as it provides resistance to the attack of pathogens and diseases. It has also been reported that PPO may exert a direct relationship with photosynthesis, as it assists in the maintenance of system homeostasis (Boeckx et al., 2015). This enzyme performs the oxidation of diphenol in quinone, beneficial compounds for the photosystem. In addition, during this process, PPO removes excess O_2_ from the system, avoiding the possible formation of superoxide radicals (Boeckx et al., 2015).

In this way, the results found in this work, besides being related to the direct effects of the amino acids in the plant metabolism, are also related to the indirect effects of signaling that they provide when exogenously applied. However, there is still no information to prove the relationship among amino acids, GLRs, and signaling in soybean crop.

## Conclusion

The use of glutamate, cysteine, phenylalanine, and glycine has a positive effect on the antioxidant metabolism of soybean plants, whether applied to seed, leaves or both. This increased the activity of anti-stress enzymes (CAT, POD, and SOD) and resistance enzymes (PAL and PPO), besides a reduction of LP.

In conclusion, a management practice that may be promising is the application of the glutamate + cysteine + phenylalanine + glycine to soybean leaves.

## Author Contributions

WT and LS wrote the main manuscript text and performed the experiments. EF and DN conceived and designed the experiments. WT executed the analyses. RU, DN, EF, LS, and KR revised the manuscript.

## Conflict of Interest Statement

The authors declare that the research was conducted in the absence of any commercial or financial relationships that could be construed as a potential conflict of interest.
